# Evaluating the environmental preferences and spread of the invasive Asian Jumping Worm ( *Amynthas spp.)* in southwestern Virginia

**DOI:** 10.17912/micropub.biology.002000

**Published:** 2026-04-08

**Authors:** Adeline Gaudet, Ashley Jernigan

**Affiliations:** 1 Department of Natural Resources and Environment, Virginia Polytechnic Institute and State University, Blacksburg, VA, US; 2 School of Plant and Environmental Sciences, Virginia Polytechnic Institute and State University, Blacksburg, VA, US

## Abstract

Asian Jumping Worms (AJW) are an invasive species in North America that can negatively impact soil habitats. This study investigated AJW environmental preferences, movement over time, and overwintering ability in Virginia, USA. Worms were sampled on four dates, two in fall 2024 and two in spring 2025, in four areas with different environmental conditions: (1) well-drained, shady (2) well-drained, mid-day sun (3) moist, shady (4) well-drained, early sun. Results indicate AJW may have delayed invasions in mulched landscapes and a preference for well-drained soil. These findings increase our understanding of AJW environmental patterns, which may help stop their spread.

**Figure 1. Abundance of Asian Jumping Worms and non-Asian Jumping Worms:** (A) Bars represent the number of Asian Jumping worms (mean ± SE) found in each block by sampling date. Bars with the same letter are not significantly different (P > 0.05). (B) &nbsp;Bars represent the number of non-AJW (mean ± SE) per quadrat in each block averaged over all four sampling dates. Bars with the same letter are not significantly different (P > 0.05). (C) Table indicating the total number of both AJW and Non-AJW in every block for each every sampling date (1 = 10/18/2024, 2 = 11/1/2024, 3 = 3/27/2025, 4 = 4/8/2025).

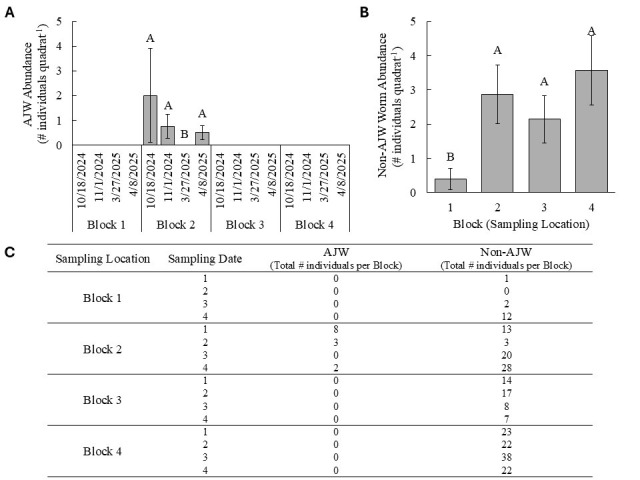



## Description

Earthworms in the genus *Amynthas* (family Megascolecidae) (NCBI Taxonomy ID: 195544), which originate from East and Southeast Asia, are widespread and ecologically influential across landscapes in their native range (Chang et al. 2009). These worms often thrash aggressively when disturbed, earning their common name of Asian Jumping Worms (AJW) (Snyder 2022). AJW are highly mobile, fast growing, reproduce asexually, and they often appear in high densities due to their ability to quickly reproduce. Their high chance of invasion is likely due to their ability to shift their diet depending on availability (Laushman 2017).

AJW have existed in the United States for decades, but there is no definite location or time recognized as the first sighting. The first official record of these invasive worms was in Maryland in 1939, but it was theorized to have been present decades prior (Schult 2016).&nbsp; AJW have recently begun notably spreading through Virginia, but their distribution throughout northeastern North America is widely unknown. So far there have been records of identification in at least 29 states, encompassing at least sixteen *Amynthas* species (Reynolds 2018). The harm AJW are potentially causing due to this invasion is relatively unknown compared to other invasive worm species (Johnson 2021).

The spread of AJW is thought to be due to the transportation of horticultural materials or due to the release of fishing bait (Snyder 2022). Landscaping practices are seen as a probable cause of their spread since AJW can thrive in mulch (Snyder 2022). The worms are highly invasive in hardwood forests, urban parks, residential yards, greenhouses, and compost piles. The AJW cocoons are small, allowing them to be unknowingly spread through footwear, soils, and mulch (Ziter 2021).

AJW are responsible for altering soil habitats and reducing food resources for native flora and fauna. They can effectively remove litter layers and change the structure of the soil surface (Snyder 2022). These effects of AJW can impact the growth of a forest understory which can increase erosion and decrease forest health. AJW can reduce native biodiversity and are seen as a threat to native species, for example species like salamanders and millipedes (Ziter 2021). There are currently no effective methods to reduce AJW populations without harming other species (Ziter 2021).

This study investigated AJW environmental preferences, movement over time, and ability to survive over winter in southwestern Virginia, USA. Mulch application, a known cause of AJW spread, occurred on the study site on October 7 ^th^ , 2024. The study site was the mulched area of the Virginia Tech agriculture quad, which is landscaped with hardwood trees and ornamental bushes, and surrounded by turfgrass and sidewalks. Two sampling events occurred in the fall shortly after mulch application (October 18 ^th^ , 2024 and November 1 ^st^ , 2024) and two the following spring (March 27 ^th^ , 2025 and April 8 ^th^ , 2025). Four blocks (4 m x 4 m) were established in the mulched area that had different environmental conditions: (1) well-drained, shady (2) well-drained, mid-day sun (3) moist, shady (4) well-drained, early sun. Four randomly placed 0.25 m ^2^ quadrats were assessed within each block by sorting through the mulch and disturbing the soil to a depth of 7 cm to identify and count earthworms.

Over the four sampling dates, AJW were only found during the 1 ^st^ , 2 ^nd^ , and 4 ^th^ samplings. The AJW were affected by the block and sampling date interaction ( *P* =<0.001, npar = 9), and were only found in Block 2, the well-drained with mid-day sun area ( Figure 1A & Figure 1C ). We also found that the non-AJW abundance was greater than the AJW abundance. The non-AJW abundance was greater in Blocks 2, 3, and 4 compared to Block 1 ( *P* = 0.012, df = 12) ( Figure 1B & Figure 1C ).

Since the AJW were only found in block 2, it is likely that this area was the original location of the AJW introduction. &nbsp;The AJW did not spread from their initial point of introduction during the duration of this study, which may suggest that their environmental preferences in mulched landscapes are well-drained areas. Research shows that when leaf litter or mulch is dry, the worms tend to prefer the topsoil, but when it is wet, they prefer to reside in the leaf litter (Chang et al., 2021). &nbsp;During this study AJW were only found in the topsoil and not in the mulch itself, which is likely due to the dry conditions during the sampling events.

&nbsp;More likely, the lack of AJW spread from the initial point of introduction may have been due to an invasion delay or the inhospitable nature of block 1. Block 1 was in an area with high foot traffic and was adjacent to a manhole cover. The spread of the AJW could have been deterred by the conditions in block 1, therefore effectively preventing them from continuing their spread across the agriculture quad. Block 1 had the fewest number of worms overall, further demonstrating that it was not a suitable habitat for any worm species. Non-AJW were found in high numbers in all blocks, except block 1, which could mean that the presence of Asian Jumping worms did not impact the population of Non-Asian Jumping worm species.

AJW were present in Spring, indicating that they were able to survive over winter in Virginia in the mulched landscape. Adults die at the beginning of winter but produce cocoons that are frost-hard and can remain viable at field temperatures as low as −24&nbsp;°C (Görres et al., 2016). They were not found in the first spring sampling but were present two weeks later during the second spring sampling. Since their life cycle is one year and the cocoons are produced during early winter, the cocoons only hatch when soil conditions are considered favorable (Chang et al., 2021). AJW can tolerate a wide temperature range, with viability between 20 and 26&nbsp;°C, though exposure above 38.4&nbsp;°C kills the AJW (Johnston and Herrick, 2019). Other worms were already present as adults while the AJW were still likely cocoons or juveniles due to cold conditions observed in early Spring 2025. In the northeastern United States, juvenile AJW typically appear in late March or early April once soil temperatures reach approximately 10 °C (Blackmon et al., 2019) and remain above the minimum survival threshold of 5 °C (Richardson et al., 2009).

AJW are not a new invasive species in the United States, but there is limited research and understanding of their ecology and prevention methods. There is no federally approved method of removal for AJW due to the lack of research and understanding of this species. We inferred that their environmental preferences in southwest Virginia are well drained and warm with a limited spread in human inhabited areas. For an effective prevention method to be introduced, we must continue to further understand their ecology in different climates and habitats.

## Methods

*Experiment Design*

A field experiment was conducted to understand the environmental preferences and spread of *Amynthas spp.* The study area was the agriculture quad (37°13'31.2"N 80°25'25.7"W) on Virginia Tech’s campus in Blacksburg, Virginia, USA. Triple black walnut mulch was applied on October 7 ^th^ , 2024 to the study area using air pressured blowers. In this experiment, the four blocks were established (4 m x 4 m) that represented different environmental conditions: block 1 was well drained and shady, block 2 was well drained and was received mid-say sun, block 3 was shady and moist, and block 4 was well drained and received early morning sun. Two sampling events occurred in the fall shortly after mulch application (October 18 ^th^ , 2024 and November 1 ^st^ , 2024) and two the following spring (March 27 ^th^ , 2025 and April 8 ^th^ , 2025). Sampling occurred in the morning on each date to standardize the results.

*Worm Presence Assessment*

Within each block, four 0.25 m ^2^ quadrats were randomly sampled at each sampling date. For each quadrat, a hand trowel was used to move the mulch from the quadrat into a separate container, without disturbing the soil beneath the mulch. After the mulch was removed from the quadrat, the top 7 cm of soil were quickly and carefully disturbed to assess worm presence. Any worms present, both AJW and non-AJW were recorded. Any unidentifiable worms were placed in a labeled container to further ID in the lab using stereomicroscopes and taxonomic keys (Chang et al., 2016; OPAL, 2016). The AJW found were likely a multispecies group which coexist: *A. agrestis* (NCBI Taxonomy ID: 2613858), *A. tokioensis* (NCBI Taxonomy ID: 585707), and *Metaphire hilgendorfi* (NCBI Taxonomy ID: 506675). The remaining mulch was shifted through to assess worm presence. Any worms, both AJW and non-AJW were recorded. As the mulch was sifted, it was placed back in the correct quadrat.

*Data Analysis*

Data analyses were performed in R version 3.4.2 (R core team, 2017). An ANOVA was used to test differences in AJW counts using the g *lmer* function in the ‘glmmTMB’ package using a Poisson distribution to account for the zero inflated data. Since AJWs were only found in Block 2, model convergence was not reached. Paired T-tests were instead conducted in Excel to compare AJW abundance in Block 2 between the sampling dates.

An ANOVA was used to test differences in non-AJW worm counts using the *lmer* function in the ‘lme4’ package. The non-AJW worm abundance data was log +1 transformed to meet the assumptions of normality and homoscedasticity for the ANOVA. The sampling location (block), sampling date, and their interactions were included as fixed effects, and a random quadrat nested in block effect was included to account for potential variability due to randomized quadrat placement in each block for both models. Pairwise mean comparisons were made by using Tukey adjustment, and significance was declared for *P* ≤ 0.05.
